# Surgical treatment algorithms for post-burn contractures

**DOI:** 10.1186/s41038-017-0074-z

**Published:** 2017-03-14

**Authors:** Kenji Hayashida, Sadanori Akita

**Affiliations:** 1 Division of Plastic and Reconstructive Surgery, Department of Dermatology, Faculty of Medicine, Shimane University Hospital, 89-1 Enya-cho, Izumo, Shimane 693-0021 Japan; 2 Department of Plastic Surgery, Wound Repair and Regeneration, Fukuoka University, Fukuoka, Japan

**Keywords:** BurnContracture, Algorithm, Donor site morbidity, Perforator flap, Surgical treatment

## Abstract

Burn contractures produce restrictions in motion and unacceptable aesthetic results, frequently with persistent wounds. Proper planning and tissue selection are essential to minimize donor site morbidity optimizing outcomes. The principle of burn reconstructive surgery requires that the defects after release should be replaced with donor tissues which have matching texture and color as well as enough pliability. Autologous skin grafting or flap surgeries meet these criteria to replace scar tissues and resurface the subsequent to post-released scar defects. Despite the benefits, the use of flaps is often limited in burn patients for many reasons. If a surgeon intends to release completely and reconstruct in one-stage operation, a large defect may result in large donor site morbidity, necessitating flap surgery including free flap surgery. A lot of different methods and procedures are available for resurfacing the defects, and these are reviewed. In this article, algorithms for the release of burn contractures and reconstructive methods are presented. These treatment algorithms should aid in achieving significant improvement in both joint motions and aesthetic deformities.

## Background

Burn injury is still the common cause of trauma especially in low- and middle-income countries [1]. Deep partial-thickness and full-thickness burns that are not treated with early excision and grafting can be disabling, as these deep injuries often lead to burn scar contractures unless provided with adequate positioning and splinting. Burn scar contractures are severely disfiguring, painful, and itching. As such thing, patients with burn scar contractures which interfere with activities of daily living are often marginalized and experience difficulties in receiving education and securing work [2].

There are a number of therapies to reduce contractures including intra-lesional corticosteroid injection, antihistamines, hydrotherapy, dynamic or static splinting, laser therapy, compression therapy, and surgical excision and reconstruction; yet, it is still unknown which therapy should be chosen for which contracture, when they should be initiated, and how long should be the period or how often they should be continued [3–6].

Generally, contractures arise where adequate burn care has not been applied. Even though scar management has been instigated in a vigorous manner, the contracture may also occur secondary to split-thickness skin grafting to the burn wounds. Another point, the contracture does not only occur due to skin loss but also may result from the differential growth pattern between burn scar and surrounding tissues [7].

The most powerful treatment option for contracture release is a surgical procedure. The defect should be replaced with the donor tissues matching texture, color, and pliability. Skin flaps including free flaps meet these criteria to replace scar tissues and repair the resulting defect post release, providing superior functional outcomes [8–10]. Indeed, the gold standard for burn scar reconstruction is to use adjacent skin flaps to minimize differences in skin characteristics. However, achieving a balance between scar resurfacing and minimizing donor site morbidity is a challenging problem that depends on the size of the area involved, the region of involvement, and the availability of the non-scarred tissue for use as skin flaps. Many surgical treatments are available for burn scar contracture release. However, a recent systematic review showed that it is still unclear which surgical procedure is the most effective [11]. Thus, surgeons are facing with clinical problems. The purpose of this review will be to outline the use of skin grafts, flaps, and devices currently used in burn scar contracture as well as provide insight into flap surgery that might make a future impact in burn patients.

## Review

### Principles of surgical contracture release and reconstruction

Generally, release of burn contracture is considered once the scar forming of the contracture is thought to be matured. This is based on the conventional idea that interfering with an active scar will lead to further contracture formation. This waiting approach is representative of what the contracture release and split-thickness skin grafting was the most widely performed procedure until recently. If split-thickness skin grafting is applied to a wound, this wound would contract with the potentiality of recurrent contracture again. Additional procedures would be required to normalize subsequent contractures. These include physiotherapy to mobilize the joints and splints to preserve the range of motion (ROM) [12]. Since contracture and hypertrophic scarring increase up to the first 6 months, patients should be followed up frequently.

Recent studies and articles have questioned this waiting period. As an example, full-thickness skin grafting has been shown to reduce the incidence of subsequent ectropion in the acute phase of lower eyelid burns [13]. The important thing is that this timing restriction is not capable of being applied when the defects are planned to be covered by full-thickness skin or flap [14]. De Lorenzi et al. [15] have reported waiting for 2 or 3 weeks acutely, prior to considering release and free flap covering, and their success rates of 94%. The procedures provided good functional and aesthetic results with low morbidity on both acute deep burns and delayed reconstructions.

As described in a number of articles [16, 17], flap surgeries are preferable to a full-thickness skin graft. Besides, the concept of “perforator flap” allows us to harvest thinner flaps like full-thickness skin [18]. Tissue that does not re-contract and will grow with the patients should be used for the release of scar contractures. For this purpose, locally available tissue is preferred because it provides tissue of superior quality and contains healthy adjacent skin and subcutaneous adiposal tissue. A first study published in 2017 compared the perforator-based interposition flaps and the full-thickness grafts in the management of burn scar contractures [19]. The open randomized controlled trial revealed that perforator-based interposition flaps resulted in a more effective scar contracture release than full-thickness skin grafts. They observed an increase in surface area of the flaps to 123 percent after 3 months and a further increase to 142 percent after 12 months. In contrast, full-thickness skin grafts showed a significant contraction; the remaining surface area decreased to 87% after 3 months and 92% after 12 months. The versatility and safety of local flaps has been improved by incorporating perforator vessels, because the vascular supply is secured and perforators are located in throughout the body. Perforator-based propeller flaps and so-called ad hoc perforator-based flaps provide well-vascularized skin and soft tissue which has reliable pliability [20, 21]. As the multicenter randomized study excluded scars of the face and scalp, it seems that these methods are especially effective in reconstruction of burn contracture of extremities and trunk to preserve ROM across joints.

### Surgical treatment options

#### Extremities

**Fig. 1:**
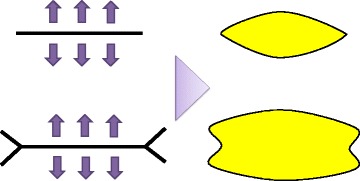
Inadequate incision cannot release the contracture sufficiently. Bilateral fish-tailed incision should be made at appropriate points

**Fig. 2:**
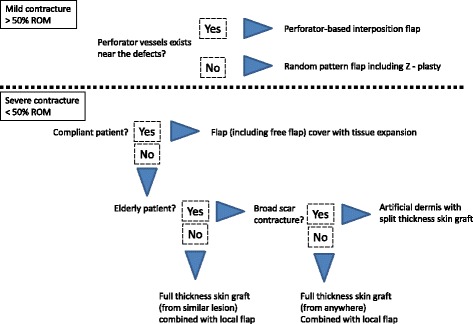
In the extremities, an algorithm for the defect after release of burn scar contracture

The contracture must be fully released whether a band or a sheet of scar tissue exists. The incision for contracture release should be done at the meridian of the joint and fish-tailed at both ends of the scar, extending into normal medial tissue and lateral tissue (Fig. 1). This procedure will increase the size of the defect to be covered. Then, reconstructive procedures include the following (Fig. 2).

##### In mild contractures (>50% joint range of motion)

**Fig. 3:**
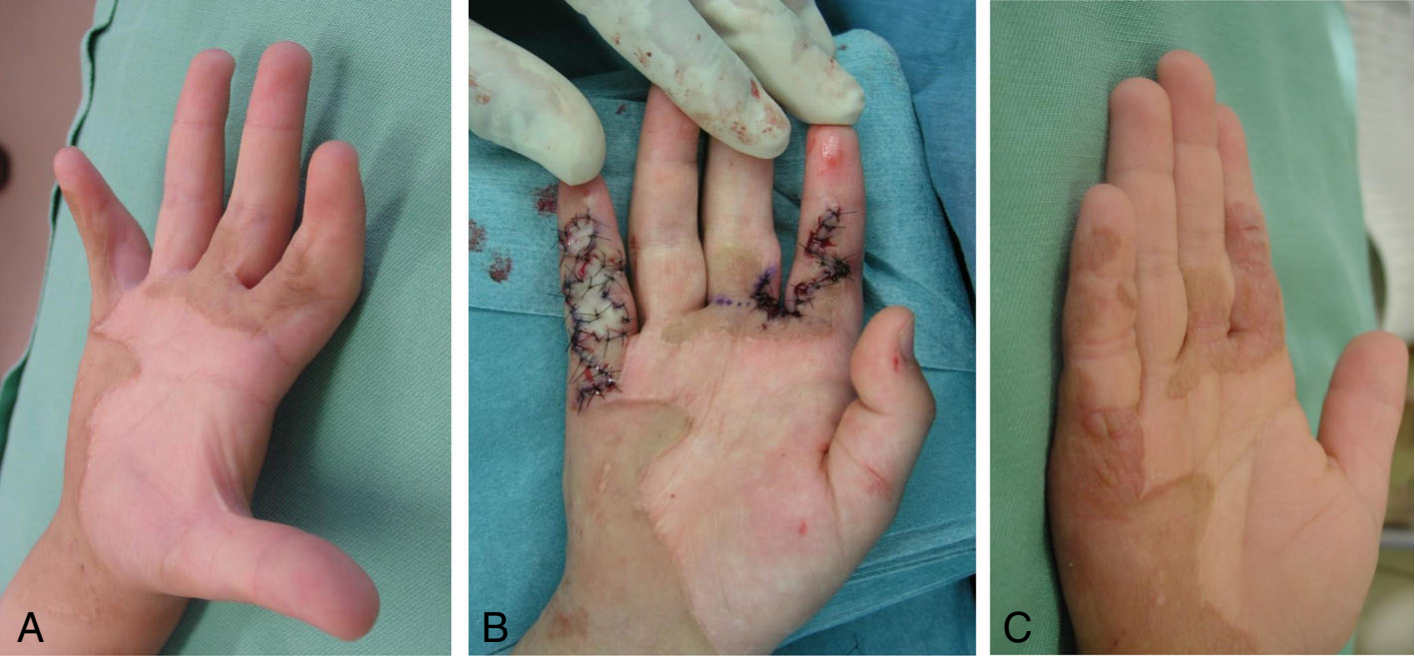
**a** and **b**. Contracture of index finger volar surface with Z-plasty. Release of little finger volar contracture with full-thickness skin graft from planter region of the foot. **c**. One year after reconstructive surgery

For extremity contracture, Hudson et al. divided them into mild and severe type by less or greater than 50% ROM on the basis of their much experience in this field [14]. The classification is a simple judging method to determine the severity of post-burn contractures. Local tissue rearrangement such as Z-plasty can be used to lengthen and transpose the scar. Transposing the flaps of Z-plasty lengthens the central limb and narrows the involved scar by the medial transposition of the flaps. And then, the flap tips should be incised perpendicular to the central limb for a short distance to supply more enough skin and soft tissue. Following transposition, the irregular borders could help to camouflage the scars (Fig. 3). Many variations of Z-plasty and YV-plasty including the opposite running YV-plasty [22] have been described such as W-plasty, 5 or 4 flap-plasty, and multiple Z-plasty. When the scar around a band is mature, these variations are more easily applied. Unless the scar is mature, these local tissues around inflammatory scars may result in flap necrosis.

##### In severe contractures (<50% joint range of motion)

When there is not sufficient adjacent tissue to allow the surgeons to release or lengthen the contracture, very diverse surgical approaches are needed. After the release of a contracture, reconstruction with full-thickness skin grafting has a better texture or color match than split-thickness skin grafting, and is associated with less recurrence. The more dermis in the graft, the less contracture is demonstrated. However, in case of extensive burn, full-thickness skin graft is not practical to cover the large areas [23]. Available skin to harvest is limited (Fig. 3). Besides, the graft especially from groin area will usually show hyperpigmentation which should impair the aesthetic result.

A desirable option is flap covering. Both local flaps including propeller flaps and free flaps including pure perforator free flaps have been successfully used in burn contracture reconstruction [24–33]. For a small defect, we recommend an algorithm that was previously published by Verhaegen et al. [34]. In this algorithm, a perforator-based flap is pedicled resulting in a non-islanded flap, meaning that the skin base is left intact. If the vascularization of the flap appeared to be compromised in the intended position, the flap was converted to an islanded flap. This was a clinical decision that allowed for greater angles of rotation where necessary. Low recurrence rate is the most important advantage of the flap transferring. The wide variety of flap choice allows the surgeon to make judgement depending upon each individual case. One limitation of perforator-based local flaps is that sufficient adjacent normal skin has to be available. In that case, free flap transfer is a good option especially in the extremities, when only one joint has a severe contracture from broadened sheet of scar. Since perforator vessels are usually protected in most burn cases, the vessels can be used as recipient vessels for free flap transfer. However, free flaps frequently result in importing tissue of different color, texture, and thickness. This may lead to unacceptable aesthetic result. Furthermore, the flap must be the same size as the defect. This should lead to a large donor defect requiring coverage with split-thickness skin graft.

#### Face

**Fig. 4:**
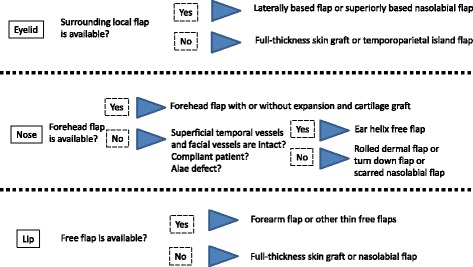
In the face, an algorithm for the defect after release of burn scar contracture

The long-term results showed confidently that flaps perform better than full-thickness skin grafts in providing a safe and effective method to resurface post-burn scar contractures in the face. Understandably, the aesthetic results are better as well. However, a randomized controlled trial for the scars of the face has not been performed to determine which technique has the best effectiveness in post-burn contracture release. Besides, because of the relative lack of objective data on outcomes, individualized reconstructions pertaining to each unique aesthetic region of the face should be performed. The most problematic late outcomes that Philip et al. identified after facial burns included gaps between grafts and hairline, eyelid ectropion, nose asymmetry, and marked hypertrophic scarring around the lip [35]. In our algorithms, we chose these three areas in the face to improve the post-burn patients’ quality of life (Fig. 4).

##### Eyelids

Late complications of eyelid burns include ectropion and lagophthalmos as a result of secondary burn contractures [35]. Significantly, this may result in further corneal damage and risk to sight [36]. However, total visual loss is thankfully rare. The goal in management of eyelid burn contractures is preservation of eyesight, prevention of future complications, and aesthetic restoration [37]. There are a number of operative procedures and methods for managing periorbital burn scar contractures. To correct ectropion, the upper eyelid should be released at the level of the supra-tarsal fold, and the lower eyelid should be released at the sub-ciliary margin. And then, they are reconstructed using full-thickness skin grafts or local flaps such as laterally based cutaneous flaps or reverse flow nasolabial flaps [38]. However, the surrounding tissue is usually not available because of severe burn scarring, in addition to the added facial scarring. Therefore, temporoparietal fascial flap based on temporal arteries in periorbital reconstruction has been widely accepted including eyebrow reconstruction with hidden scars [39]. The island flap including a reverse flow flap provides an optimal result for the eyelid contracture with minimal donor site morbidities. Elbanoby TM [40] described two skin paddles based on a single superficial temporal artery to reconstruct the upper and lower eyelids simultaneously. There were some cases whose flap may be hairy, but the patients can undergo laser hair ablation to correct and solve this issue. This technique may be innovative and useful for the reconstruction of three different unique aesthetic facial units (eyebrow, upper eyelid, and lower eyelid) with one-stage island flap.

##### Nose

Secondary nasal reconstruction is based on an assessment of the residual functional and aesthetic problems. Airway narrowing or nasal valving are managed using internal nostril splints and standard rhinoplasty principles. The forehead flap with or without expansion is usually used for reconstruction of non-graftable nasal defect. However, it is difficult for local flap options to be used because the adjacent tissue may be damaged as well as eyelid reconstruction. The forehead is often involved in a full-thickness burn, and conchal cartilage may be limited for graft material. As such thing, there are a number of articles of the use of free flaps to reconstruct the nose [41]. Reconstruction of the nasal tip, columella, and alae by free flaps derived from the base of the helix seems to give good functional and aesthetic results. The structural similarities between the nasal alar and the auricular helix have allowed the use of free helical composite grafts to reconstruct small nasal defects of less than 2.0 cm. Although relatively easy to perform technically, they are restricted by problems such as viability, dimensions, and atrophy. A free tissue transfer harvested from the ascending helix has been used to repair defects of the distal part of the nose [42]. As a non-microsurgical technique, based on the well-documented anatomy of the superficial temporal vessel, Li et al. described a microvascular auricular flap by a reversed superficial temporal vessels pedicle to surgically treat the distal defects of the nose [43]. The presence of vascular communications between the anterior frontal branch of the superficial temporal system and the supraorbital and supratrochlear arterial systems allowed this flap to be used in a reverse blood flow fashion. Krastinova et al. described a simple and reliable procedure [44]. They reported their experience of nasal alar reconstruction by a scar tissue remodeling technique using a rolled dermal flap with overlying full-thickness skin graft according to aesthetic units in a single operation. Taylor et al. have used an inferiorly based nasal turndown flap in 28 patients [45]. In their article, contraction of local scar tissue created bulk and support for nasal tip and alar, eliminating the need for distant tissue transfer or cartilage grafting. The flap consists of the dorsal surface of the nose, usually made up of skin graft and scar. Dissection is carried down to the periosteum with care to preserve a broad blood supply. The flap is then folded down on itself, and the resulting defect is resurfaced with a medium to thick split-thickness skin graft. This simple technique has been outstandingly successful for the post-burn patients with nasal deformities.

##### Lip

The lip deformity after burn injury may be complex such as a functional disfigurement interfering with eating, airways, and speech, and/or an appearance. Hypertrophic scar contracture of the lip and chin area frequently causes eversion of both the upper and lower lip. There is an intrinsic lip contracture which cannot be corrected by a neck scar release alone. Also, associated scarring at the oral commissures may limit the ability to open the mouth. In that case, sufficient skin and soft tissue are needed by transplant to the lip and chin area. A lot of reconstructive methods for burn deformities and defects of the lips have been developed [46–51]. The effective technique Saadeldeen WM [46] introduced for cheiloplasty in burn scar management creates natural lip lines and natural lip red substance without any aggressive undermining or adding any more scars. His surgical technique for upper lip cheiloplasty was performed as follows: excision of the scarred Cupid’s bow and upper lip vermilion lifting to augment the vermilion and redraw the lazy M-shaped Cupid’s bow. Lower lip cheiloplasty was designed for bordering, using a full-thickness skin graft for the chin area. After 12 months follow-up, the end result was assessed by patient satisfaction, which was fairly good in 89.1% of cases. However, it needs skin graft. The take rates of full-thickness skin graft around the mouth should be low, significantly because of difficulties of complete rest and prevention from food and fluid. Regarding the lip reconstruction in burn cases, free flap covering based on an aesthetic unit is frequently better than local flaps in the aspect of its texture. Reconstruction of labio-mental sulcus relatively seems to be difficult. For a free flap, the thickness of the flap should be reduced mostly and the cervico-mental angle tends to be dull, causing unnatural contour as well. Forearm flap and other thin flaps can solve these problems. In flap setting, Lee JW [47] defatted the forearm flap base partially, and incision was done to the dermis about 2–3 cm in length. Then, they adhered the dermis of the defatted flap to the lower lip muscle to produce an acceptable sulcus. Oh SJ [51] described a hairy preauricular free flap to correct a moderate defect of the upper lip for male. The flap was harvested from hairy posterior sideburn skin of preauricular area including superficial temporal vessels. The procedure resulted in an acceptable appearance with normal hairy skin.

### Growth factors: surgical applications to prevent scar contracture

Growth factors are endogenous signaling proteins intimately involved in wound healing. These proteins are upregulated in response to tissue damage and act through autocrine, paracrine, or endocrine mechanisms to facilitate re-epithelialization by binding to membrane-bound or cytoplasmic receptors. Even at low concentrations, growth factors can have a profound impact on the wound microenvironment, leading to rapid increases in cell migration, proliferation, and differentiation. Application of human-recombinant growth factors has been shown to mimic these effects, allowing for external modulation of the healing process. This has led to a number of applications in the surgical area where controlled delivery of growth factors holds great therapeutic potential. In fact, perioperative delivery of exogenous growth factors is a routine adjunctive treatment in a number of surgical specialties, including burn surgery.

Growth factors are classified into several families based on their characteristics. The most relevant growth factor families for wound healing are the epidermal growth factor (EGF), transforming growth factor β (TGF-β), platelet-derived growth factor (PDGF), vascular endothelial growth factor (VEGF), and fibroblast growth factor (FGF).

In terms of scarring, some articles demonstrated that FGF-treated scars showed a better process of skin remodeling, which may avoid the subsequent development of fibro-proliferative disorders [52–57]. The FGF family contains more than 20 members, of which, the most relevant for wound healing are FGF-2, FGF-7, FGF-10, and FGF-22. FGF-2, also known as basic FGF, is released from damaged endothelial cells, macrophage, or monocyte and is one of the most potent isoforms in FGF family. The bFGF is a mitogenic and chemotactic factor for fibroblasts and endothelial cells, and stimulates angiogenesis. In burn treatment, accelerated wound healing, maintenance of the complex system of melanization, and diminishing activity of erythema by bFGF will lead the wound to a closer pliability and color to adjacent skins [53, 57]. Besides, bFGF treatment is effective to prevent scar contracture especially in pediatric palmar burns [58]. While current results are promising, additional clinical trials are needed before FGF becomes widely accepted in surgical management for burn patients to avoid scar contractures and hypertrophic scars.

### Use of artificial dermis

Artificial dermis as a scaffold has been used and developed in reconstructive surgery, and now we can see some reports of one-stage skin grafting with it [59, 60]. There are several reports that this material is effective in skin defects, which are accompanied with exposure of deep structures and more brittle local tissue textures [61]. Artificial dermis allows for neodermis to form over the surface of the wound and minimizes the number of migrating myofibroblasts. Besides, combined use of bFGF is furthermore effective in order to facilitate the formation of good granulation tissue and to reduce postoperative contraction [62]. This resulted in less contraction with fewer adhesions to the underlying bones, tendons, and nerves. However, dermal substitution in post-burn surgery has not become practical, because a subjective and objective long-term follow-up study showed that no significant differences were found for skin elasticity, scar contraction, Vancouver Scar Scale, and patient’s impression in both categories between a combination of the collagen substitute with an autograft and the conventional split-thickness autograft [63].

Disadvantages include donor site morbidity from split-thickness skin graft harvesting, lower take rates than conventional autografts, and higher cost implications. Usually, it is a two-stage procedure. Soejima et al. found that areas of keloid cannot respond to artificial dermis and tend to lead to re-contracture [64]. Despite adequate splinting, the results of artificial dermis over the joints are on the whole disappointing [65]. For these reasons, treatment of burn deformity with artificial dermis may be beneficial in selected cases including elderly patients with a poor systemic condition.

However, recent advances in the field of tissue engineering and dermal substitution may create new optimal alternative methods for post-burn scar reconstruction in the near future. Stem cell technology is one of the candidates toward the treatment with artificial dermis. Among these stem cells, adipose-derived stem cells (ADSCs) can be harvested with a minimally invasive method by liposuction through small incisions. Akita et al. showed that ADSCs mixed with fat tissue have potential in cell therapy together with an artificial dermis for neck contracture. In the article, the injected subcutaneous lesion has still kept its soft texture and demonstrated the thick and vascularized soft tissue in 6 months after cell therapy [66].

### Use of tissue expanders

The reconstruction using stage transfer of expanded thin flaps is a relatively safe method. Expanded skin is the same or very close in color, thickness, and texture as adjacent skins. The procedure using tissue expander is commonly performed in the neck, chest, and scalp. Also, the use of pre-expanded scapular free flaps is a practicable procedure to ameliorate the final aesthetic appearance and functional result for the reconstruction of neck contracture by reducing donor site morbidity [67]. Gao et al. [68] reported the expanded flap of “super-thin flaps.” The advantages of this type of flaps are the following:

1. Very large flaps can be harvested because of the expansion
2. Super-thin flaps can be employed with safety
3. Texture and color match are good in the case of the anterior chest wall near the recipient site
4. Donor sites can be closed primarily
5. Microsurgical operation is not required

However, in the burned extremity, on the contrary, the tissue expander seems to be difficult to use. This procedure requires two or three operations, and the multiple surgeries should be stressful for patients. Also, it is often difficult to predict the size of the defect at the time of organizing the expansion. Infection, leakage, and skin ischemia may be complicated by expansion. There is often a considerable delay between diagnosis and treatment for the complications, as multiple operations are required. Compliant and perseverant patients are needed, and multiple patients’ visits are required to inflate the expanders weekly or so.

In contrast to full-thickness skin grafts, flaps in the expanded tissue contain subcutaneous adipose tissue. It may be hypothesized that the subcutaneous fat acts as a functional gliding layer to prevent the skins from adhering to the underlying tissue. In addition, the presence of subcutaneous adipose tissue may also explain the fact that the flaps are found to be more similar to normal skin and softer than full-thickness skin.

## Conclusions

Full-thickness skin grafts and local flaps produce satisfactory outcomes when performed for the reconstruction of burn contracture defects by experienced surgeons. Free perforator flap transfer with minimal morbidities must be useful in patients who are willing to receive a longer operation and a longer hospital stay with the benefit of improved aesthetic results at both recipient and donor site. In the future, interposition flaps based on a perforator may become a mainstay in post-burn extremity and trunk surgery.

Beneficial algorithms have been invented which are used to assist reconstructive surgeons in selecting appropriate reconstructive methods following release of burn scar contractures. These algorithms are an attempt to simplify the approach to burn contracture reconstruction. Advantages and disadvantages of these different modalities should be considered and compared before deciding the treatment.
